# Levels of vascular endothelial growth factor-A_165b_ (VEGF-A_165b_) are elevated in experimental glaucoma

**Published:** 2008-08-18

**Authors:** Ceren Ergorul, Arjun Ray, Wei Huang, Diane Darland, Zhonghui K. Luo, Cynthia L. Grosskreutz

**Affiliations:** 1 Howe Laboratory of Ophthalmology, Massachusetts Eye and Ear Infirmary, Harvard Medical School, Boston, MA; 2 University of North Dakota, Department of Biology, Grand Forks, ND

## Abstract

**Purpose:**

Although ischemia has previously been suggested to contribute to the pathogenesis of glaucoma, neovascularization is not implicated in glaucoma. Because vascular endothelial growth factor-A (VEGF-A) is a key mediator in neovascularization response, we investigated the levels of the major pro-angiogenic (VEGF-A_164_) and anti-angiogenic VEGF-A subtypes (VEGF-A_165b_) in the retina during experimental glaucoma.

**Methods:**

Glaucoma was induced unilaterally in rats by injecting 1.9 M hypertonic saline solution in the episcleral veins. The contralateral eye served as the control. The intraocular pressure (IOP) of each eye was measured via Tonopen in conscious rats. Eyes were enucleated either on the 5th or the 10th day of elevated IOP. Whole retinal lysates were separated by SDS–PAGE and transferred to PVDF membranes. Levels of VEGF-A_164_ and VEGF-A_165b_ were analyzed by western blotting using specific antibodies. In a different group of rats, retinal ganglion cells were retrogradely labeled by injecting Fluorogold in the superior colliculus a week before the induction of glaucoma. After the eyes were enucleated on the fifth day of elevated IOP, posterior eye cups were sectioned using a cryostat. Levels and localization of VEGF-A_164_ and VEGF-A_165b_ were examined in retinal sections by immunohistochemistry.

**Results:**

VEGF-A_164_ levels remained unchanged between the control and glaucomatous retinas after five days (p=0.341) and 10 days of elevated IOP (p=0.117). The presence of the anti-angiogenic VEGF-A isoform has not been previously reported in the rat. An antibody specific to VEGF-A_165b_ detected the anti-angiogenic protein in the rat retina. VEGF-A_165b_ levels were significantly increased (2.33±0.44 fold, p=0.014) in the glaucomatous retinas compared to those in controls after five days of elevated IOP. VEGF-A_165b_ levels were not different (p=0.864) between the control and glaucomatous retinas following 10 days of elevated IOP. Expression of both VEGF-A_164_ and VEGF-A_165b_ were observed in the retinal ganglion cells (RGC) and inner nuclear layer (INL).

**Conclusions:**

Five day elevation of IOP leads to an increase in the anti-angiogenic VEGF-A_165b_ levels but not in the pro-angiogenic VEGF-A_164_ levels in the glaucomatous retina. VEGF-A_165b_ levels return to baseline after 10 days of elevated IOP, and VEGF-A_164_ levels remain unchanged. We speculate that the short-term elevation of VEGF-A_165b_ levels and/or the unchanged levels of VEGF-A_164_ contribute to the lack of neovascularization in the glaucomatous retina.

## Introduction

Glaucoma is a neurodegenerative disease of retinal ganglion cells (RGC) that leads to blindness. Although the most prominent risk factor for RGC death in glaucoma is elevated intraocular pressure (IOP), the sequence of events by which IOP causes RGC death still remains largely unknown. One possible mechanism is that elevated IOP can induce abnormalities in blood flow in the glaucomatous eye. In open-angle glaucoma patients, abnormal vascular autoregulation has been observed in the inferior temporal retinal artery, the central retinal artery, the circulation of the optic nerve head, the choroid, and the perifoveal macular capillaries [1-8]. It has been suggested that dysregulation of blood flow may lead to decreased vascular perfusion in the retina and in the optic nerve head, resulting in an hypoxic response [9,10].

In the classical view of hypoxia, the ischemic tissue compensates for a decrease in oxygen levels by forming new blood vessels, a process known as neovascularization [11]. VEGF-A is a key mediator in neovascularization in ischemic retinopathies [12-14]. There are several VEGF-A isoforms expressed from a single gene via alternative splicing [15,16]. Among these, VEGF-A_165_ is the most abundantly expressed pro-angiogenic isoform in the retina [17]. More recently, anti-angiogenic sister isoforms of VEGF-A have also been identified [18-20]. For example, VEGF-A_165b_, an anti-angiogenic human VEGF-A isoform, has been shown to inhibit VEGF-A induced neovascularization in the mouse retina following ischemia [21].

There are only a few studies that have examined VEGF-A in glaucoma. VEGF levels were shown to be increased in the plasma of glaucoma patients when compared to that of healthy controls [22] and in the aqueous humor of glaucoma patients when compared to their plasma VEGF levels [23]. Despite these findings, neovascularization is not implicated in glaucoma, and the role of VEGF-A has not been examined in the glaucomatous retina.

If ischemia contributes to the pathogenesis of glaucoma, why is there no neovascularization in glaucoma? To answer this apparent paradox, we investigated the levels of pro-angiogenic VEGF-A_164_ (the rat version of VEGF-A_165_) and anti-angiogenic VEGF-A_165b_ (the rat version of VEGF-A_165b_) in normal and glaucomatous retinas after a short-term (five day) and an intermediate-term (10 day) elevation of IOP. Because of the lack of neovascularization in glaucoma, we hypothesized that the levels of VEGF-A_165b_ but not VEGF-A_164_ would be increased in the glaucomatous retina.

## Methods

### Subjects

Male rats (retired breeder Brown Norway; 300-450 g; n=16) were used for the study. Rats had ad libitum access to food and water during the study and were kept on a 12 h illumination cycle. All animal related procedures were performed in accordance with the statement for the use of animals in research released by the Association for Research in Vision and Ophthalmology.

### Retrograde labeling of retinal ganglion cells

Rats (n=4) were anesthetized with an intraperitoneal injection of 1.5 mg/kg of acepromazine maleate, 7.5 mg/kg of xylazine, and 75 mg/kg of ketamine (Webster Veterinary Supply, Sterling, MA). Following shaving of the head, each rat was placed in a stereotaxic instrument. The skin covering the skull was incised along the midline using a surgical blade, and the skull was exposed and leveled. Next, for each hemisphere, a 30-gauge stainless steel needle was lowered into the superior colliculus at 5.3 mm posterior to the bregma, 1.5 mm lateral to the midline, and 4.8 mm ventral to the skull surface. Using a 5 μl syringe (Hamilton, Reno, NV), 2 μl of Fluorogold solution (3% in PBS with 10% DMSO; Fluorochrome, Denver, CO) was injected over 10 min into each hemisphere. Following the injections, the skin was sutured. Rats were allowed to recover for a week before glaucoma was induced experimentally.

### Experimental induction of glaucoma

To elevate IOP, hypertonic saline solution (1.9 M) was unilaterally injected in the episcleral veins as described by Morrison and colleagues [24]. The contralateral eye of the rat served as the control. A maximum number of three injections that were two weeks apart were performed in the absence of IOP elevation. Rats that did not have an elevation of IOP after the third surgery were excluded from the study.

### Intraocular pressure measurements

IOPs were measured with a TonoPen XL tonometer (Medtronic Ophthalmics, Jacksonville, FL) in conscious rats [25]. Measurements were taken between 10 AM and 2 PM. Before the first hypertonic saline injection, baseline IOPs for both eyes were measured for each rat. Following glaucoma inducing surgery, IOPs were measured three times a week. On each measurement day, an average of 15 readings was calculated for each eye. This study investigated a five day (n=6) and a 10 day elevation of IOP (n=6).

### Tissue preparation

Rats were sacrificed by CO_2_ inhalation either after five days or 10 days of elevated IOP. For western blotting, retinas were isolated from eyes obtained after five days and 10 days of elevated IOP (six pairs each). Retinas were placed in 200 μl of 1 mM of EDTA/EGTA/DTT, 10 mM of Hepes (pH=7.6), 0.5% Igepal (Sigma Chemical Co., St. Louis, MO), 42 mM of KCl, 5 mM of MgCl_2_, 1 mM of PMSF, and a tablet of protease inhibitors (Complete Mini, Roche Diagnostics, Mannheim, Germany). After retinas were sonicated and incubated for 15 min on ice, samples were spun at 21,000 rpm at 4 °C for 30 min. Retinal proteins were quantified by spectrophotometry using the Bio-Rad D_c_ Protein Assay (Bio-Rad Laboratories, Hercules, CA).

For immunohistochemistry, four pairs of eyes that were enucleated after five days of elevated IOP were fixed with 4% paraformaldehyde for 20 min at room temperature. These eyes were previously back-labeled with Fluorogold. Next, the posterior eye cups were isolated and fixed with 4% paraformaldehyde for an additional 40 min at room temperature. After posterior eye cups were cryoprotected overnight in graded sucrose dilutions, they were placed in the optimal cutting temperature compound (Tissue-tek, Miles Diagnostic Division, Elkhart, IN) and were sectioned 16 μm thick using a cryostat.

### Western blotting

Retinal proteins isolated after either five days or 10 days of elevated IOP were separated on Tris-HCl Ready-Gels (Bio-Rad Laboratories, Hercules, CA). Recombinant rat VEGF-A_164_ protein (25–250 ng; R&D Systems, Minneapolis, MN) was also loaded as a positive control in certain experiments. Proteins separated by SDS–PAGE were then transferred to polyvinylidene difluoride membranes (Immobilon-P; Millipore, Billerica, MA) for 1 h. After the membrane was blocked for 1 h at room temperature with 2% ECL Advance Blocking Agent (GE Healthcare, Piscataway, NJ) in Tris-buffered saline with Tween (TBS-T), it was incubated at 4 °C overnight with a primary antibody. The primary antibodies used in this study were as follows: rabbit polyclonal anti-VEGF (1:50; Santa Cruz Biotechnology, Santa Cruz, CA), mouse monoclonal anti-VEGF165B (1:1,000; Abcam, Cambridge, MA), and mouse monoclonal anti-α-tubulin (1:100,000; Sigma, Saint Louis, MO). The next day, membranes were incubated for 1 h at room temperature with peroxidase-conjugated secondary antibodies. Goat anti-rabbit IgG (1:10,000–1:40,000; Jackson ImmunoResearch, West Grove, PA) and goat anti-mouse IgG (1:20,000–1:100,000; Jackson ImmunoResearch) were the secondary antibodies used in the study. Both of these antibodies had minimal cross-reaction to rat serum proteins. Next, membranes were processed with ECL Advance Western Blotting Detection Kit (GE Healthcare, Piscataway, NJ) and exposed to Kodak BioMax Light Film (Crestream Health, Inc., Rochester, NY).

### Densitometry and statistical analysis

A Personal Densitometer SI (Molecular Dynamics, Sunnyvale, CA) was used to scan the exposed films. The density of the protein of interest on the film was measured using ImageQuant 1.2 (Molecular Dynamics). First, the background density was subtracted from the density of each band. Next, the densitometric reading of the protein of interest was normalized to α-tubulin readings, which served as loading controls. For each retina pair, the normalized densitometric reading from the glaucomatous retina was divided by the reading from the control retina. Then, the ratios from different pairs of retinas were averaged. For statistical analysis, a one-sample *t*-test was used to evaluate the significance of the ratios for a given protein (one-tailed, hypothesized mean=1, α level=0.05). Data were reported as mean±standard error of the mean (SEM) in the text.

### Immunohistochemistry

Retinal sections from four pairs of eyes were blocked for 1 h at room temperature in 4% normal goat serum and 0.3% Triton-X 100 in 1X PBS, pH 7.4. Sections were incubated with the primary antibody overnight at 4 °C. Some sections were incubated in blocking solution without the primary antibody and were used as negative controls. The primary antibodies and the dilutions used in the study were mouse monoclonal anti-VEGF (20 μg/ml; Sigma) and mouse monoclonal anti-VEGF165B (1:500; Abcam). The following day, sections were incubated with the Alexa Fluor 594-conjugated goat anti-mouse secondary antibody (1:500; Invitrogen, Carlsbad, CA) for 1 h at room temperature. After the sections were treated with Prolong Gold anti-fade reagent, staining was visualized using an Olympus BX51 microscope (Olympus, Center Valley, PA).

On our BX51 microscope, we used UPlanApo 0.70 NA 20X (Olympus) and UPlanApo 0.85 NA 40X (Olympus) objective lenses through a 10X ocular or camera lens to image our retinal sections for a total magnification of 200X or 400X, respectively. Excitation/emission filter cubes used for a given fluorophore were 11006v2 Gold (Chroma, Rockingham, VT) for Fluorogold and N41004 HQ Texas Red (Chroma) for Alexa Fluor 594. We used DPController 1.2.1.108 (Olympus) in conjunction with the DP70 color camera (Olympus) affixed to the BX51 to image the retinal sections for fluorescence. We then used Adobe Photoshop to layer the images and apply transparency to see the overlap of different fluorophores in a given section area.

## Results

### Levels of VEGF-A_164_ do not change in the glaucomatous retina

Average peak IOP was (mean±SEM) 40.7±1.9 mmHg and 43.1±0.8 mmHg for the five-day (n=6) and 10-day (n=6) groups that were used in the western blot (WB) analysis, respectively. Average peak IOP was 43.4±0.8 mmHg for the other five-day group used for the immunohistochemistry (IHC) analysis (n=4).

**Figure 1:**
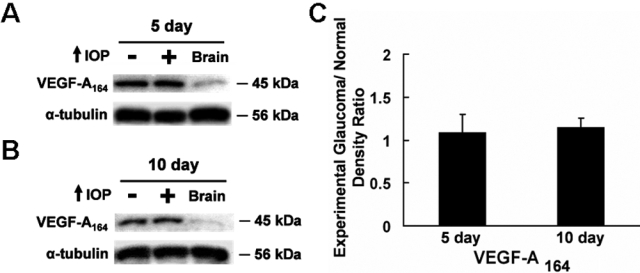
Western blot analysis of VEGF-A_164_ expression in the glaucomatous retina. **A**: VEGF-A_164_ was detected at 45 kDa in control and glaucomatous retinas after five days of elevated IOP. **B**: Similarly, VEGF-A_164_ was observed at 45 kDa in the control and glaucomatous retinas following 10 days of elevated IOP. **C**: Glaucomatous/control ratio of normalized VEGF-A_164_ densitometry readings in the retina is demonstrated in the chart. VEGF-A_164_ was expressed at comparable levels in the control and glaucomatous retinas after five and 10 days of elevated IOP. The positive control was the brain, and the loading control was α-tubulin.

In western blots, anti-VEGF antibody detected a 45 kDa band corresponding to the VEGF-A_164_ dimer in all retinas and in the positive control brain (Figure 1). VEGF-A_164_ levels remained unchanged between the control and glaucomatous retinas after five days (p=0.341, n=6; Figure 1A,C) and 10 days of elevated IOP (p=0.117, n=6; Figure 1B,C). Using this well characterized antibody [26,27], the VEGF-A_164_ monomer was not detected in the retina or in the brain.

**Figure 2:**
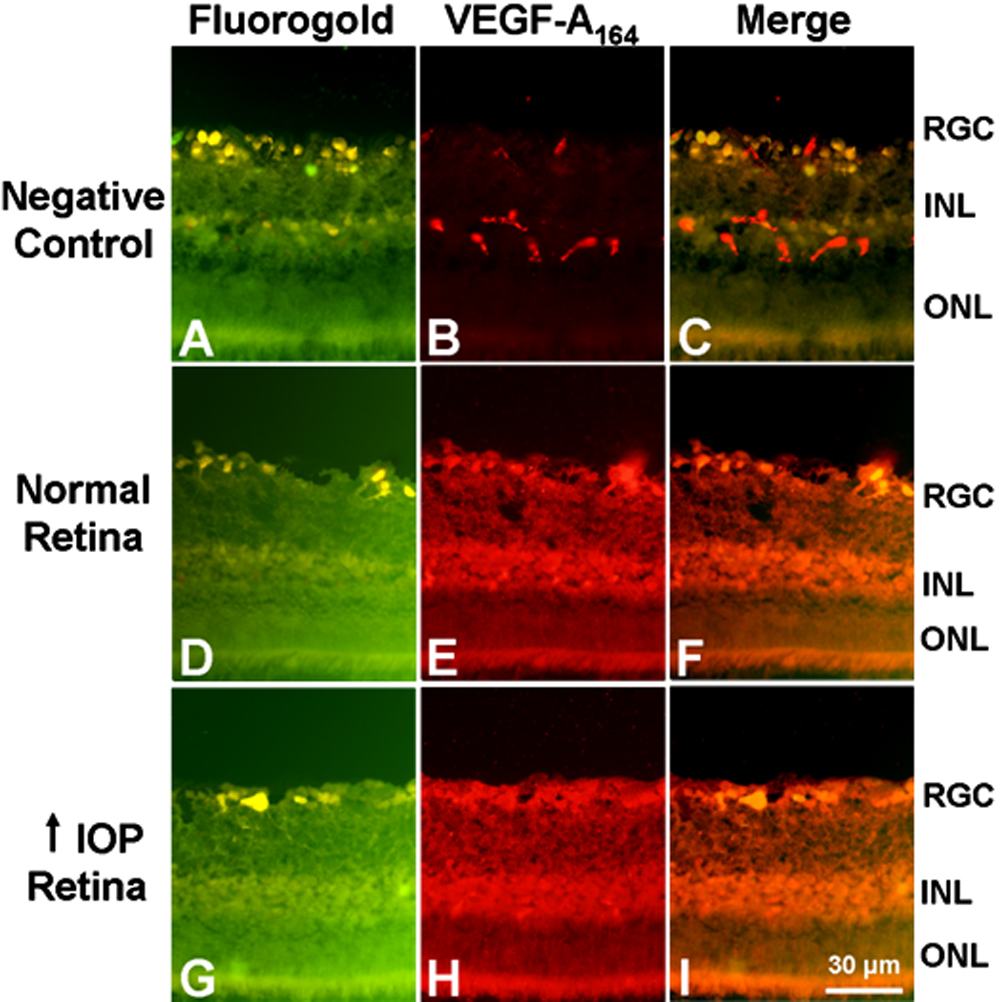
Immunohistochemical analysis of VEGF-A_164_ expression in the glaucomatous retina after five days of elevated IOP. **A**-**C**: Negative control. Some non-specific staining of blood vessels in the RGC and the INL was observed. **D**-**F**: VEGF-A_164_ staining of the normal retina (n=4). VEGF-A_164_ was present in the RGC and the INL. **G**-**I**: VEGF-A_164_ staining of the glaucomatous retina (n=4). Staining was detected in the RGC and INL. VEGF-A_164_ levels did not differ between the normal and glaucomatous retinas.

In VEGF-A_164_ IHC, there was some nonspecific staining in the blood vessels in the RGC layer and INL of the negative control retinas (Figure 2B,C). VEGF-A_164_ staining did not differ between the normal (Figure 2E) and glaucomatous retinas (Figure 2H). VEGF-A_164_ expression was localized to the RGC and the cells in the INL of both groups (Figure 2E,H). In the RGC layer, VEGF-A_164_ staining colocalized with the retinal ganglion cell marker, Fluorogold (Figure 2F,I). Also, VEGF-A_164_ levels did not differ between the normal and glaucomatous retinas, which are consistent with the WB results.

### Anti-VEGF-A_165b_ antibody does not recognize VEGF-A_164_

**Figure 3:**
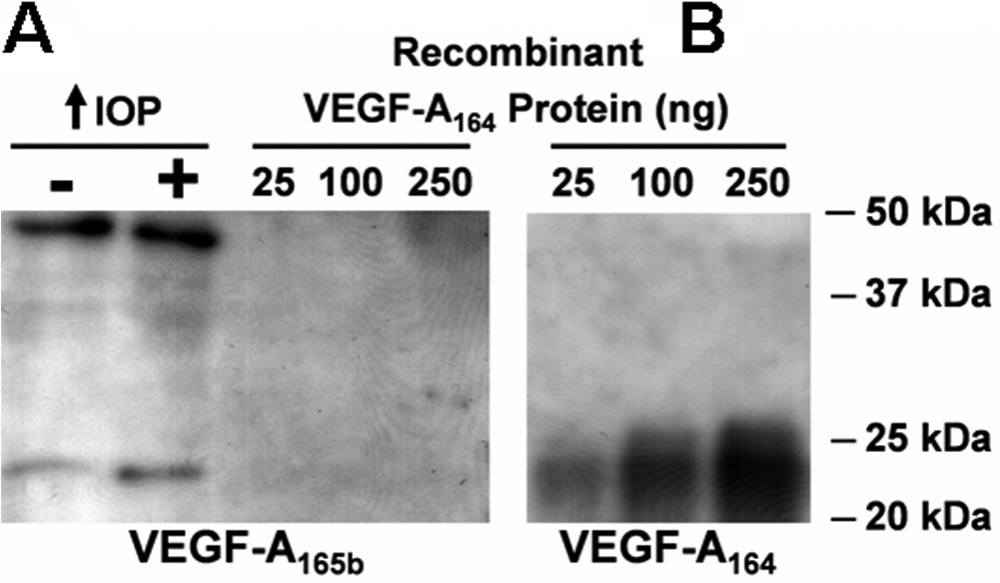
Anti-VEGF-A_165b_ antibody does not recognize VEGF-A_164_*_._* **A**: Incubation with VEGF-A_165b_ antibody. This antibody recognizes the VEGF-A_165b_ monomer (22.5 kDa) and dimer (45 kDa) in control and glaucomatous retinas (first two lanes). VEGF-A_165b_ antibody does not recognize 25 ng, 100 ng, or 250 ng of VEGF-A_164_ recombinant protein in the same membrane (last three lanes). **B**: Incubation of the same membrane with the anti-VEGF antibody after stripping. The anti-VEGF antibody recognizes VEGF-A_164_ recombinant protein at all concentrations.

Because it had been predicted that VEGF-A_164_ and VEGF-A_165b_ are highly homologous in amino acid sequence [18], we first investigated whether the anti-VEGF-A_165b_ antibody would also recognize VEGF-A_164_. To test this possibility, we immunoblotted different concentrations (25 ng, 100 ng, and 250 ng) of the recombinant rat VEGF-A_164_ protein with the anti-VEGF-A_165b_ antibody. Whereas the anti-VEGF-A_165b_ antibody did not recognize the VEGF-A_164_ protein at any concentration, it recognized two bands around 22.4 and 45 kDa in a pair of control and glaucomatous retinas corresponding to the monomer and dimer forms of VEGF-A_165b_, respectively (Figure 3A). Next, we stripped the membrane and subsequently immunoblotted with the anti-VEGF antibody. A 22.4 kDa VEGF-A_164_ monomer was detected at all concentrations (Figure 3B), confirming the presence of VEGF-A_164_ recombinant protein in the same membrane. The dimer form was not observed with the recombinant rat VEGF-A_164_ protein. The combination of these findings indicated that the anti-VEGF-A_165b_ antibody does not recognize VEGF-A_164_ and that VEGF-A_165b_ is expressed in the rat retina.

### VEGF-A_165b_ levels are increased in the glaucomatous retina

**Figure 4:**
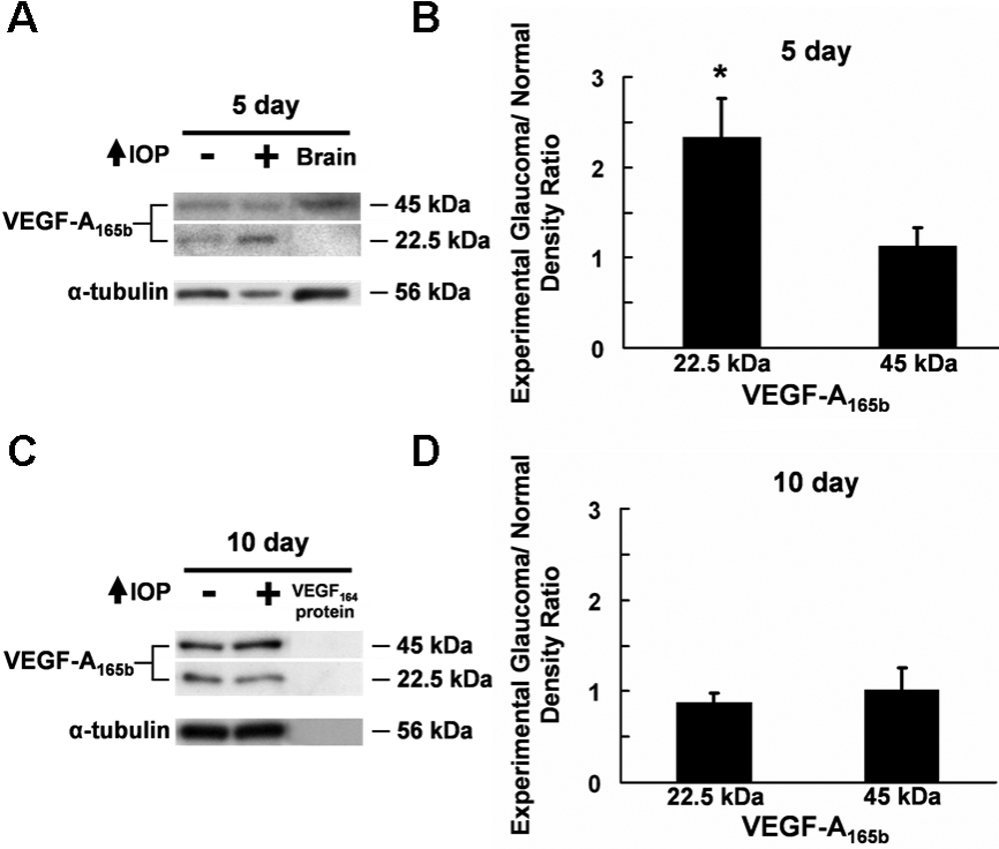
Western blot analysis of VEGF-A_165b_ expression in the glaucomatous retina. **A**: VEGF-A_165b_ expression following five days of elevated IOP. Retinal VEGF-A_165b_ monomer and dimer were detected at 22.5 and 45 kDa, respectively. **B**: Glaucomatous/control ratio of normalized VEGF-A_165b_ densitometry readings in the retina following five days of elevated IOP. Expression of the 22.5 kDa VEGF-A_165b_ was increased significantly in the glaucomatous retinas compared to the controls. **C**: VEGF-A_165b_ expression following 10 days of elevated IOP. VEGF-A_165b_ monomer and dimer were observed at 22.5 and 45 kDa in the retina, respectively. **D**: Glaucomatous/control ratio of normalized VEGF-A_165b_ densitometry readings in the retina following 10 days of elevated IOP. Both 22.5 kDa and 45 kDa VEGF-A_165b_ were expressed at comparable levels in the control and glaucomatous retinas. The positive control was the brain while the negative control was VEGF-A_164_ recombinant protein. The loading control was α-tubulin.

Following five days of elevated IOP, the anti-VEGF-A_165b_ antibody detected bands around 22.4 and 45 kDa in all retinas, which represent the monomer and dimer forms of VEGF-A_165b_, respectively (Figure 4A). Whereas VEGF-A_165b_ dimer levels remained unchanged between the control and glaucomatous retinas (p=0.273, n=6), VEGF-A_165b_ monomer levels were significantly increased in the glaucomatous retinas compared to those in controls (2.33±0.44 fold, p=0.014, n=6) (Figure 4A,B). However, following 10 days of elevated IOP, there was no change in levels for the VEGF-A_165b_ dimer (p=0.483, n=6) or for the VEGF-A_165b_ monomer (p=0.864, n=6) between the control and glaucomatous retinas (Figure 4C,D). These results indicate that VEGF-A_165b_ levels increase after five days of elevated IOP and return to baseline levels after 10 days of elevated IOP.

**Figure 5:**
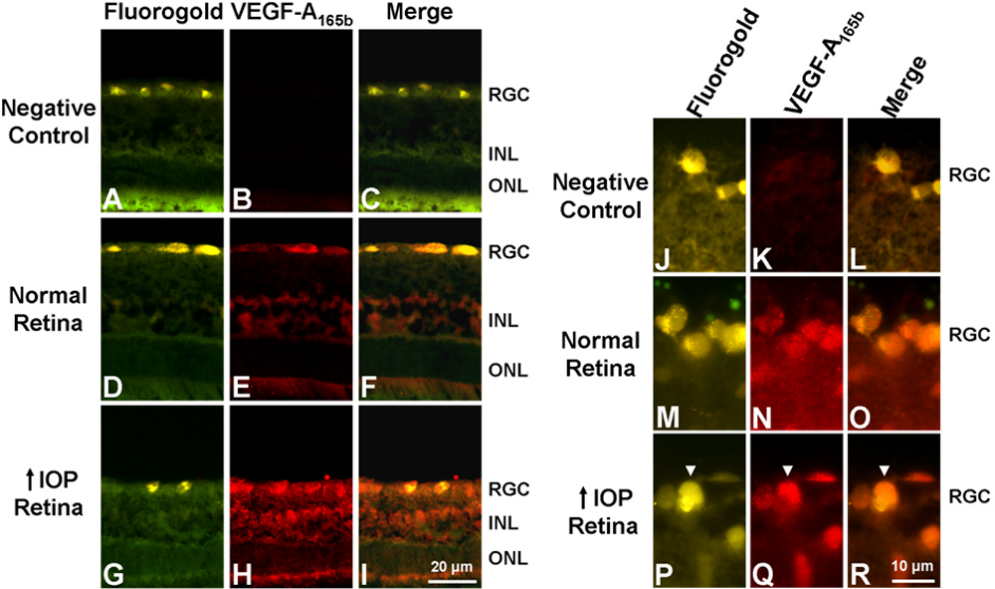
Immunohistochemical analysis of VEGF-A_165b_ expression in the glaucomatous retina after five days of elevated IOP. **A**-**C**: Negative control. Non-specific staining was not detected in the retina. **D**-**F**: VEGF-A_165b_ staining of the normal retina (n=4). VEGF-A_165b_ was present in the RGC and the INL. **G**-**I**: VEGF-A_165b_ staining of the glaucomatous retina (n=4). VEGF-A_165b_ staining was stronger in the RGC and the INL compared to the normal retina. **J**-**L**: Negative control. No non-specific staining was observed in the retina. **M**-**O**: VEGF-A_165b_ staining of the RGC in the normal retina. Staining was colocalized with the RGC marker, Fluorogold. **P**-**R**: VEGF-A_165b_ staining of the RGC in the glaucomatous retina. Levels of VEGF-A_165b_ in the Fluorogold-labeled RGC (white arrow) were increased in the retinas with elevated IOP.

IHC demonstrated that the distribution of VEGF-A_165b_ was similar to that of VEGF-A_164_. VEGF-A_165b_ staining was observed in the RGC and in the inner nuclear layer (Figure 5E,H). In the RGC layer, staining overlapped with the RGC marker, Fluorogold (Figure 5F,I,O,R). No staining was observed in the negative control with the primary antibody omitted (Figure 5B,K). Consistent with our WB results, IHC analysis showed increased levels of VEGF-A_165b_ immunoreactivity in the glaucomatous retinas compared to normal retinas (Figure 5H,Q verses Figure 5E,N, respectively).

## Discussion

We demonstrate in this report that VEGF-A_165b_ is present in the rat. In particular, we show that VEGF-A_165b_ is present in the retina and localized primarily to the RGC layer and the inner nuclear layer. Our findings for VEGF-A_165b_ show a distribution similar to that seen for VEGF-A_164_ in this report and previous reports about VEGF-A_164_ by others [28,29]. Using back labeling techniques, we find that RGC express VEGF-A_165b_. Our data show that VEGF-A_165b_ levels are increased early in the cause of experimental glaucoma but return to baseline at a later time point. IHC results show that this increase is primarily due to increased expression in the RGC layer and in the INL.

Our results demonstrate that the levels for the pro-angiogenic VEGF-A_164_ do not change in the glaucomatous retina compared to control retinas in the rat after five days or 10 days of elevated IOP. Consistent with previous studies, we observe that VEGF-A_164_ is expressed in the RGC and INL of the retina [28,29].

VEGF-A_165_ is the most abundantly expressed pro-angiogenic isoform in the retina [17]. Both VEGF-A_165_ and VEGF-A_165b_ mRNA are produced from the VEGF-A pre-mRNA via alternative splicing [15,18]. VEGF-A_165_ and VEGF-A_165b_ share a 96.4% homology and differ only in the last six amino acids in their amino acid sequence in humans [18]. However, while VEGF-A_165_ is pro-angiogenic, VEGF-A_165b_ has an inhibitory effect on angiogenesis both in vitro and in vivo [18,19]. For example, VEGF-A_165b_ inhibits neovascularization in the mouse retina following oxygen-induced retinopathy [21]. More recently, other inhibitory splice variants of VEGF-A have also been identified [19,20]. It has been suggested that the relative levels of the pro-angiogenic and anti-angiogenic VEGF-A isoforms determine whether angiogenesis will be stimulated or inhibited in a tissue [20]. For instance, the expression of the pro-angiogenic VEGF-A isoforms increases in the vitreous of human patients with diabetic retinopathy whereas the expression of the anti-angiogenic VEGF-A isoforms remains unchanged compared to the normal vitreous [20]. Among the anti-angiogenic VEGF-A isoforms, VEGF-A_165b_ is observed to be the dominant isoform [15,18].

What molecular mechanism is responsible for the upregulation of VEGF-A_165b_ mRNA in the glaucomatous retina? Although the precise answer remains unknown, proposed mechanisms include differential promoter selection, alternate regulation of mRNA stability, and regulation of alternative splicing [15,30-33]. In alternative splicing, as the *VEGF-A* gene is being transcribed, the emerging pre-mRNA is instantaneously processed by several RNA-binding proteins and splice factors [15]. These proteins bind to the auxiliary sequences on the pre-mRNA and determine which exons will be spliced [34]. It is thought that several signal transduction pathways, which are activated in response to changes in the environment (e.g., receptor-mediated pathways, neuronal activity, cellular stress-like hypoxia) affect alternative splicing by altering the relative levels of RNA-binding proteins and splice factors or the localization of splice factors within the cell [15,35,36]. More recently, microRNAs have also been shown to alter alternative splicing [37]. For example, in muscle and neuronal development, microRNAs lead to the inclusion of alternative exons by suppressing a repressor protein of alternative splicing [38,39]. In addition, transcriptional events may also affect the regulation of alternative splicing. For instance, the speed of RNA polymerase II can influence the choice of splice sites and recruitment of regulatory factors [40].

In conclusion, we report an increase in the retinal levels of the anti-angiogenic VEGF-A_165b_ but not the pro-angiogenic VEGF-A_164_ in our experimental glaucoma model. The combination of these findings suggests that the elevation of VEGF-A_165b_ levels and/or the unchanged levels of VEGF-A_164_ contribute to the lack of neovascularization in the retina in glaucoma.
